# The T2-FLAIR Mismatch Sign as an Imaging Indicator of IDH-Mutant, 1p/19q Non-Codeleted Lower Grade Gliomas: A Systematic Review and Diagnostic Accuracy Meta-Analysis

**DOI:** 10.3390/diagnostics11091620

**Published:** 2021-09-04

**Authors:** Antonis Adamou, Eleftherios T. Beltsios, Panagiotis Papanagiotou

**Affiliations:** 1 Department of Radiology and Medical Imaging, University of Thessaly, 41110 Larissa, Greece; antonadamou@gmail.com; 2 Faculty of Medicine, School of Health Sciences, University of Thessaly, 41110 Larissa, Greece; beltsioseleftherios@gmail.com; 3 Department of Diagnostic and Interventional Neuroradiology, Hospital Bremen-Mitte/Bremen-Ost, 28205 Bremen, Germany; 4 First Department of Radiology, School of Medicine, National & Kapodistrian University of Athens, Areteion Hospital, 11528 Athens, Greece

**Keywords:** T2-FLAIR mismatch sign, glioma, astrocytoma, diagnostic accuracy, systematic review, meta-analysis

## Abstract

The study’s objective was the evaluation of the diagnostic accuracy of the T2-FLAIR mismatch sign in terms of diagnosing IDH-mutant non-codeleted (IDHmut-Noncodel) lower grade gliomas (LGG) of the brain. We searched the MEDLINE, Scopus and Cochrane Central databases. The last database search was performed on 12 April 2021. Studies that met the following were included: MRI scan assessing the presence of T2-FLAIR mismatch sign, and available IDH mutation and 1p/19q codeletion status. The quality of studies was assessed using the QUADAS-2 tool. Twelve studies involving 14 cohorts were included in the quantitative analysis. The diagnostic odds ratio [DOR (95% confidence interval; CI)] was estimated at 34.42 (20.95, 56.56), *P_z_* < 0.01. Pooled sensitivity and specificity (95% CI) were estimated at 40% (31–50%; *P_z_* = 0.05) and 97% (93–99%; *P_z_* < 0.01), respectively. The likelihood ratio (LR; 95% CI) for a positive test was 11.39 (6.10, 21.29; *P_z_* < 0.01) and the LR (95% CI) for a negative test was 0.40 (0.24, 0.65; *P_z_* < 0.01).The T2-FLAIR mismatch sign is a highly specific biomarker for the diagnosis of IDHmut-Noncodel LGGs. However, the test was found positive in some other tumors and had a high number of false negative results. The diagnostic accuracy of the mismatch sign might be improved when combined with further imaging parameters.

## 1. Introduction

Diagnosing and treating malignant brain tumors is challenging for clinicians. Histopathology remains the gold standard for the diagnosis of such tumors. The molecular differentiation of brain tumors has become a useful guiding tool for clinicians. In 2016, WHO published the revised classification of tumors of the central nervous system (CNS), which includes the molecular profile of each tumor subtype [1].

Radiomic features are radiological parameters that may contribute in accurately diagnosing a disease, by extracting and quantifying information from medical images. Following the trend for digitalization of the information in clinical practice, radiomics represented a useful tool to support clinicians in decision-making [2]. Several radiomic features have been tested, in an attempt to differentiate between gliomas of the brain. Among them, the T2-FLAIR mismatch sign is a radiomic feature reported by multiple studies as a highly specific biomarker of diffuse astrocytomas (IDH-mutant non-codeleted lower grade gliomas; IDHmut-Noncodel LGGs) of the brain. This feature is defined by a mismatch in T2 and FLAIR magnetic resonance (MR) sequences, particularly the presence of complete/near-complete hyperintense signal on T2WI, and relatively hypointense signal on FLAIR except for a hyperintense peripheral rim [3].

Given the importance of the mismatch sign potentially implicating the non-invasive diagnosis of IDHmut-Noncodel LGGs, the present study is intended at evaluating the diagnostic accuracy of the T2-FLAIR mismatch sign in terms of diagnosing these tumors.

## 2. Materials and Methods

### 2.1. Protocol

The protocol for this systematic review and meta-analysis was registered to the PROSPERO registry for systematic review protocols (ID number: CRD42021248328) and is available in full on https://www.crd.york.ac.uk/prospero/display_record.php?ID=CRD42021248328 (retrieved 15 May 2021).

### 2.2. Literature Search

A comprehensive search of the MEDLINE, Scopus and Cochrane Central databases was conducted. The last database search was performed on 12 April 2021. The search algorithm included the following keywords combined with “AND” and “OR” Boolean operators: “radiomic features”, “T2-FLAIR”, “mismatch sign”, “glioma”. The references of each article were manually screened for eligible articles. Furthermore, the related articles section in each database was reviewed for eligible articles.

### 2.3. Inclusion and Exclusion Criteria

Studies that met both the inclusion criteria were included: (1) MRI scan assessing the presence of T2-FLAIR–mismatch sign, (2) available IDH mutation and 1p/19q codeletion status. Exclusion criteria were (1) did not include the radiomic feature of interest, (2) reviews/case reports/letters, (3) no English language, and (4) 1p/19q codeletion status not reported.

### 2.4. Data Extraction

Two authors (A.A. and E.T.B.) reviewed the titles and abstracts for eligibility. Discrepancies between reviewers were resolved by a third reviewer (P.P.). The full-text articles of the selected studies were further evaluated. Only studies that met the inclusion criteria were eventually included. Data were collected for the following variables: (1) study characteristics (author, year of publication, number of cases, median age, M/F ratio, WHO grades, and interrater agreement), (2) number of patients with T2-FLAIR mismatch sign (positive or negative), (3) IDH mutation and 1p/19q codeletion status.

### 2.5. Statistical Analysis

We used the hierarchical regression approach (hierarchical summary receiver operating characteristic, HSROC) to analyze the diagnostic data of each study [4]. The pooled diagnostic odds ratio (DOR) and its 95% confidence interval (95% CI) were used to determine the diagnostic effectiveness of the mismatch sign.

We calculated the heterogeneity of the studies using the Cochran’s Q and I^2^indices. The random effects model was applied when heterogeneity was *I*^2^ > 75% and/or *P_Q_* < 0.10. Otherwise, the fixed effects model was applied. Additionally, pooled sensitivity and specificity and their 95% CI were calculated. The diagnostic parameters of positive predictive value (PPV), negative predictive value (NPV), likelihood ratio for a positive test (LR+), and likelihood ratio for a negative test (LR-) were also calculated.

The statistical analysis was performed with the OpenMeta[Analyst] for OS X Sierra (10.12) software [5]. Finally, the PRISMA-DTA guidelines for reporting reviews and meta-analyses of diagnostic test accuracy studies were also applied (Table S1; Supplementary Material) [6].

### 2.6. Critical Appraisal and Risk of Bias Assessment

The Quality Assessment of Diagnostic Accuracy Studies (QUADAS-2) tool [7] was used to assess risk of bias for each study. The QUADAS-2 tool is publicly available at www.quadas.org (retrieved 30 May 2021).

## 3. Results

### 3.1. Search Results and Characteristics of the Included Studies

**Figure 1:**
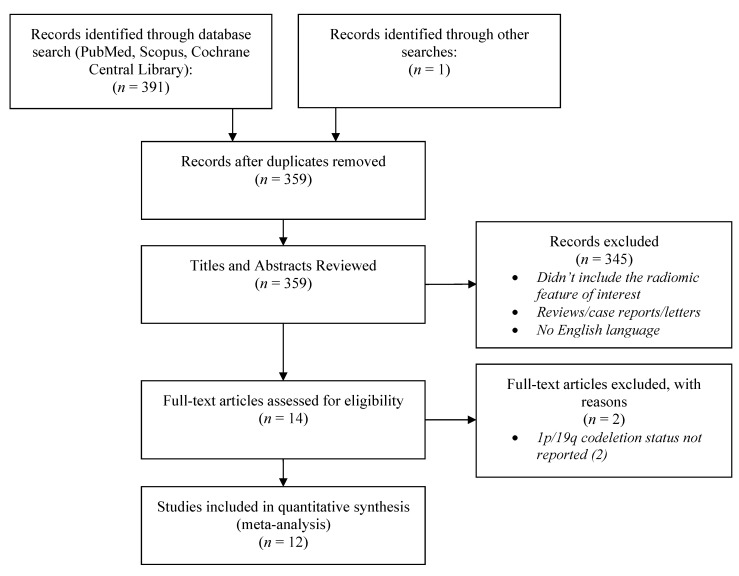
The PRISMA flow diagram of the study selection process.

The structured search of the databases and the manual screen of the references and related articles section retrieved 392 studies published between 2016 and 2021. Duplicates were removed and titles and abstracts were reviewed for their eligibility, resulting in 14 potentially eligible studies. The full-text article of each study was further reviewed. Two studies were excluded, both because they did not provide the 1p/19q codeletion status of the patients’ gliomas. Twelve studies were eventually included in the quantitative analysis, involving 1736 patients with WHO Grade II or III gliomas of the brain. Only two of the studies included cases with WHO Grade IV gliomas. In these studies, we only included the WHO Grade II and III cases in our quantitative analysis. The age of the subjects included in the present study ranged from 18 to 86 years old. The study selection flowchart according to the PRISMA guidelines is shown in Figure 1. The main characteristics of the included studies are presented in Table 1.

### 3.2. Diagnostic Test Performance for IDHmut-Noncodel Gliomas

**Figure 2:**
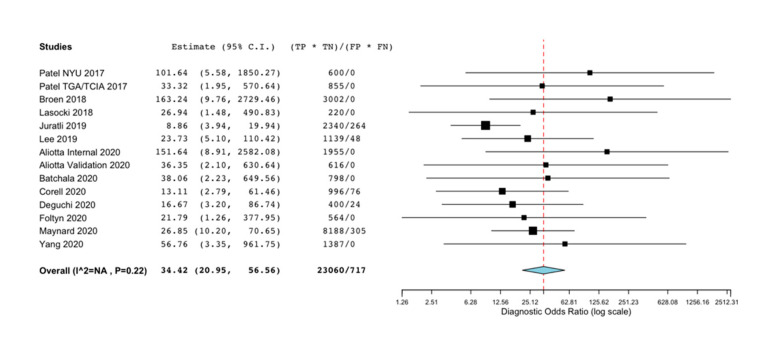
The forest plot presenting the effectiveness of the T2-FLAIR mismatch sign in diagnosing IDH mutant non-codeleted low grade gliomas.

Due to low heterogeneity (*I*^2^ = NA, *p* = 0.22), a fixed effects model (Mantel-Haenszel test) was applied to identify the diagnostic performance of the T2-FLAIR mismatch sign. The pooled quantitative analysis revealed a statistically significant linkage between the T2-FLAIR mismatch sign and the diagnosis of IDHmut-Noncodel LGGs [DOR (95% CI): 34.42 (20.95, 56.56); *P_z_* < 0.01]. The forest plot for the pooled DOR is shown in Figure 2.

### 3.3. Sensitivity and Specificity

**Figure 3:**
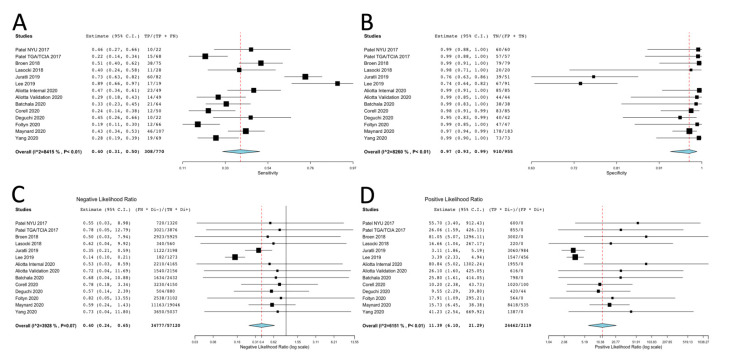
The forest plots presenting the (**A**) sensitivity, (**B**) specificity, (**C**) likelihood ratio for a negative test and (**D**) likelihood ratio for a positive test of the T2-FLAIR mismatch sign for the diagnosis of IDH mutant non-codeleted low grade gliomas.

**Figure 4:**
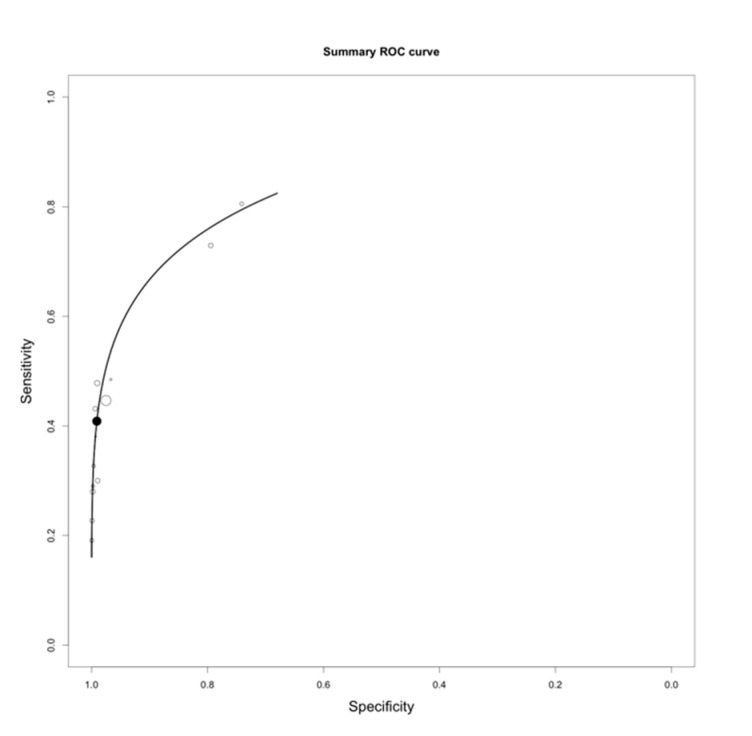
Summary receiver operating characteristic (SROC) curve of the T2-FLAIR mismatch sign’s diagnostic accuracy in diagnosing IDH mutant non-codeleted lower grade gliomas.

Due to high heterogeneity, the random effects model was applied to assess the sensitivity and specificity of the T2-FLAIR mismatch sign in diagnosing IDHmut-Noncodel LGGs. The pooled analysis yielded the following estimates (95% CI; *p*-value; *I*^2^ and Cochran’s Q indices for heterogeneity): a sensitivity of 40% (31–50%; *P_z_* = 0.05; *I*^2^ = 84.2, *P_Q_* < 0.01), and a specificity of 97% (93–99%; *P_z_* < 0.01; *I*^2^ = 82.6, *P_Q_*<0.01). Results were statistically significant for both sensitivity and specificity. The forest plots for sensitivity and specificity are presented in Figure 3. The SROC curve is demonstrated in Figure 4.

### 3.4. Likelihood Ratios for Positive and Negative Results

Due to high heterogeneity, the random effects model was used to assess the pooled LR+ of the test. Statistical analysis resulted in a LR+ of 11.39 (6.10, 21.29; *P_z_* < 0.01; *I*^2^ = 61.5, *P_Q_* < 0.01).

Due to low heterogeneity, the fixed effects model was applied to assess the pooled LR- of the test. Statistical analysis resulted in a statistically significant low LR− [0.40 (0.24, 0.65; *P_z_* < 0.01; *I*^2^ = 39.3, *P_Q_* = 0.07)].

The forest plots for pooled LR+ and LR− are shown in Figure 3.

### 3.5. Risk of Bias Assessment

We used the QUADAS-2 tool to identify possible risk of bias within the included studies. The assessment revealed low risk of bias and applicability concerns in all studies, except three [9,10,13], where the risk was high in regard to the index test, due to the assessment of the MR images by a neurologist in one study and a neurosurgeon in two studies. A high applicability concern was found in one study regarding its index test, since there was no mentioning about the observers being blinded. The results are shown in detail in Table S2 (Supplementary Material).

## 4. Discussion

The T2-FLAIR mismatch sign has been previously reported as a highly specific imaging indicator of IDHmut-Noncodel LGGs. In their meta-analysis, Goyal [19] confirmed the association of T2-FLAIR mismatch sign with the disease, with high DOR, high specificity and low sensitivity [10]. Likewise, Park et al. [20] recently reported that the sign is a highly specific, though insensitive indicator of the disease. Accordingly, the present meta-analysis reports the high diagnostic accuracy of the mismatch sign to diagnose the disease [DOR (95% CI): 34.42 (20.95, 56.56)]. We also confirm its high specificity [97% (93–99%)] and low sensitivity [40% (31–50%)] in diagnosing these groups of tumors.

The first to report the association of the sign with the diagnosis of the disease were Patel et al. [3]. The subject then attracted research attention from researchers who further studied the diagnostic accuracy of the biomarker. Lasocki et al. [8] also reported that >50% T2-FLAIR mismatch is highly predictive of non-codeleted gliomas. Furthermore, Broen et al. [9] suggested that the mismatch sign is a highly specific imaging biomarker for IDH-mutant astrocytomas. Although Juratli et al. [10] reported that the mismatch sign is 76% specific in diagnosing the disease, they thereby reported that the T2-FLAIR mismatch sign performed 96% specificity in diagnosing non-codeleted gliomas in patients younger than 40 years old at diagnosis, with a tumor larger than 6 cm in diameter. However, the sensitivity for this subgroup was even lower than the overall sensitivity. The relatively low diagnostic accuracy presented by the study, might be due to not relying on the strict imaging criteria for the mismatch sign identification that Patel et al. first suggested [21]. In another study, apart from the diagnostic accuracy of the mismatch sign, Corell et al. [13] investigated its relation to methylation profiles. They confirmed its high specificity and low sensitivity, while methylation analysis indicated that the sign does not compromise a separate subentity. The high specificity was also confirmed in a recent study [18].

The diagnosis of gliomas, in general, is and will remain histopathological. Thus, surgery is necessary to remove the tissue and study its histopathological and molecular characteristics. The discovery of radiomic biomarkers may indicate the tumor’s subtype preoperatively, or even judge the operative or non-operative nature of such a tumor. The mismatch sign is not consistently defined, since its definition varies throughout the included studies: a mismatch of >50%, >75%, or even an abstract definition of “complete or near/complete mismatch”. This inconsistency often results in disagreement between observers, although the interobserver agreement in all the included studies was moderate to high (Table 1).

The T2/FLAIR mismatch sign is reported as a highly specific biomarker, though its low sensitivity in diagnosing IDHmut-Noncodel LGGs led scientists towards combining additional parameters to improve its diagnostic performance. Batchala et al. [11] studied two retrospective cohorts, of which only the one was eligible for inclusion to the quantitative analysis of the present study. Apart from validating the high specificity of the test, they also suggested a 2-step classification algorithm based on the T2-FLAIR mismatch sign and a multivariate logistic regression model involving multiple parameters. This approach demonstrates an overall moderate prediction accuracy for 1p/19q-codeletion status in IDH-mutant LGGs [11]. Lee et al. [12] reported a specificity of 73.6% and sensitivity of 89.5%, the highest estimate for sensitivity among the included studies. They also suggested that combining the apparent diffusion coefficient (ADC) and cerebral blood volume (CBV) histogram parameters with the presence of the mismatch sign may improve the diagnostic accuracy of non-codeleted LGGs [12]. Additionally, Yang et al. [17] investigated a model for predicting the codeletion status of LGGs. They concluded that a combination of the mismatch sign, relative ADC, intratumoral susceptibility signals, and maximum relative CBV may improve the accuracy for predicting the 1p/19q codeletion status among LGGs in clinical practice.

As research interest about the mismatch sign increased, studies investigating the pathology and microenvironment of these tumors emerged. Deguchi et al. [14] reported a statistically significant association of the mismatch sign with the formation of microcysts in IDHmut-Noncodel LGGs. They further concluded that it might be common in a distinct subgroup of astrocytomas, known as protoplasmic astrocytomas. Foltyn et al. [15] reported higher ADC and lower rCBV values between gliomas with or without the presence of the mismatch sign. They also suggested potential differences in the cellularity and microenvironment within LGGs, since they present lower ADC values in the FLAIR-hyperintense rim and higher values in the hypointense interior on diffusion weighted imaging (DWI).

The high ADC values in IDH-mutant LGGs may explain the finding of microcysts reported in a previous study [14]. Aliotta et al. [16] intended to develop an ADC analysis-based approach to consistently and objectively identify IDHmut-Noncodel LGGs. Moreover, the combination of volumetric ADC and the presence of the mismatch sign for diagnosing IDHmut-Noncodel LGGs, resulted in improved sensitivity and the same specificity compared to the mismatch sign alone [16].

It is of high importance to mention that the T2/FLAIR mismatch sign may be found in other molecular subtypes, including IDH-mutant 1p/19q codeleted and IDH-wildtype gliomas. A recent case series reported five cases of false–positive mismatch signs in patients with gliomas other than 1p/19q non-codeleted LGGs [22]. Only one out of the five cases were adult, possibly indicating that age is an important factor with regard to the clinical application of the mismatch sign. The sign might be associated with tumor subtypes other than IDHmut-Noncodel LGGs in the pediatric population, thus, it might be interpreted and used cautiously in this specific subgroup [21]. Additionally, the sign is reported to be identified in dysembryoplastic neuroepithelial tumors [23], hence it is not entirely specific for IDHmut-Noncodel LGGs and should not be used as a stand-alone diagnostic imaging biomarker. Nevertheless, the sign is reported as absent in WHO Grade IV glioblastomas [15]. A recent article aiming to determine imaging metrics predictive of the IDH mutation in glioblastomas, reported fluid attenuation in non-contrast-enhancing tumors as a novel imaging biomarker, along with other conventional MRI metrics and patient age [24].

The present study is characterized by several limitations that should be mentioned. The exclusion of grey literature in our data acquisition strategy suggests possible publication bias. Thus, the results of the present analysis should be interpreted cautiously since results may be overestimated in meta-analyses including published data only [25]. Additionally, some of the included studies lacked certain information that would have allowed for testing the diagnostic performance of the mismatch sign by subgroups (age, sex, WHO grade, etc.) Furthermore, our review included retrospective studies only, with a limited number of subjects. Lastly, our study is subject to language bias since only studies written in English were included.

## 5. Conclusions

The T2-FLAIR mismatch sign is a highly specific biomarker for the diagnosis of IDH-mutant non-codeleted LGGs. However, the test has been occasionally found positive in few other tumor types, thus it has to be interpreted with caution, especially in the pediatric population. The test also reveals a high number of false negative results. The diagnostic accuracy of the mismatch sign might be improved if the imaging criteria are strictly applied or the sign is combined with further imaging parameters. Further research is needed to investigate the mismatch sign’s accuracy and its relationship with other imaging biomarkers in predicting IDH-mutant non-codeleted LGGs.

## Figures and Tables

**Table 1:** Baseline characteristics of the included studies.

Study—YOP	*n*	Mean Age	M/F Ratio	WHO Grade II	WHO Grade III	Interobserver Agreement	Method of Mismatch Identification
Patel SH et al.2017 [3]	125	45.5 (20–75)	62/63	58	67	0.73	Two neuroradiologists, both blinded to the molecular status.
Patel SH et al.2017 [3]	82	45 (21–82)	44/38	35	47	0.73	Two neuroradiologists, both blinded to the molecular status.
Lasocki A et al.2018 [8]	59	na	na	43	16	0.88	Two neuroradiologists both blinded to the molecular status.
Broen MPG et al.2018 [9]	154	43 (20–82)	86/68	133	9	0.75	One neurologist and a neuroradiologist, both blinded to the molecular status.
Juratli TA et al.2018 [10]	133	40 (18–86)	na	57	67	0.86	One neuroradiologist and a neurosurgeon, both blinded to the molecular status. A third reviewer assessed any disagreement.
Batchala PP et al.2019 [11]	102	41 (20–75)	50/52	59	43	0.56	Two neuroradiologists, both blinded to the molecular status.
Lee MK et al.2019 [12]	110	47.4 ± 13.3	56/54	45	65	ICC 89	Two neuroradiologists, both blinded to the molecular status.
Corell A et al.2020 ⊥ [13]	135	pr: 45 ±14.3retr: 47.9 ±15.7	84/56	na	na	0.77	Two neurosurgeons. A neuroradiologist resolved any disagreement. A senior neuroradiologist resolved further disagreement.
Deguchi S et al.2020 [14]	64	39 (20–65)	14/8	38	26	0.73	Two neurosurgeons, both blinded to each other’s results. A radiologist confirmed their findings.
Foltyn M et al.2020 ⊥ [15]	408	57 (47–69)	228/180	61	52	0.75	Two radiology residents and one radiologist, all blinded to the molecular status.
Aliotta E et al.2020 [16]	134	na	na	na	na	na	One neuroradiologist, both blinded to the molecular status.
Aliotta E et al.2020 [16]	93	na	na	na	na	na	One neuroradiologist, blinded to the molecular status.
Yang X et al.2020 [17]	142	39.74 ± 10.09	43/26	na	na	na	Two neuroradiologists, both blinded to tumor pathology.
Maynard J et al.2020 [18]	290	40 (33–52)	169/121	na	na	0.44–0.62	Three neuroradiologists, all blinded to diagnosis.

⊥ Studies that included WHO Grade IV cases; na: not available, YOP: year of publication, *n*: number, M/F: male/female, WHO: World Health Organization, ICC: intraclass correlation coefficient.

## Supplementary Materials

The following are available online at www.mdpi.com/article/10.3390/diagnostics11091620/s1, Table S1: Table presenting the PRISMA-DTA checklist of the study, Table S2: Table presenting the results of the QUADAS-2 tool assessment.
